# A Discourse on the Life, Character and Services of Daniel Drake, M. D.

**Published:** 1853-04

**Authors:** 


*A Discourse on the Life, Character and Services of Daniel Drake, M. D., delivered, by request, before the Faculty and Medical Students of the University of Louisville, January* 27, 1853. By S. D. Gross, M. D., Louisville. 1853. 8vo. pp. 92.

This discourse is as honorable to the heart of the distinguished author, as it is creditable to his ability as a writer. It is a tribute worthy of the character and services of the late Dr. Drake, whose decease has occasioned a void in the profession of this country, felt more sensibly in the great interior valley with which he was particularly identified by his life and labors.

The discourse is eminently a panegyric. But to this no just exceptions can be taken even by those (if there are such) whose estimate of the deceased is less exalted than that which is cherished by his enthusiastic and eloquent eulogist. Dr. Gross was long associated with Dr. Drake as a colleague and intimate friend; and while the partiality of affection would commend itself to respect rather than criticism, it is to be considered that these relations involve the best opportunites for judging correctly of character and attainments. But the evidences of the talents and industry of Dr. Drake, are before the public in his several works, and especially in the last and most elaborate publication to which he had devoted many years, and in the completion of which he was engaged at the time of his death.* An ardent and indefatigable laborer in the cause of medical education, the name of Dr. Drake will live in the recollections of the members of the numerous classes who have listened to his prelections, and in the histories of the institutions with which he was connected. True to the highest moral instincts of our nature, and to the tendencies of medical pursuits, he was not unmindful of objects of philanthropy. Aside from constant exertions, both by precept and example, in inculcating religious truth, temperance, and other virtues, we are informed by the author of this discourse that the asylum for the blind now in successful operation at Louisville, was mainly indebted to his efforts for that public interest which led to its erection and liberal endowment by the state.

In the domestic and social relations he not only was without reproach, but exemplified the devoted husband, the kind parent, the constant friend, the courteous gentleman, and the earnest Christian.

Such were the life, character and services of Dr. Drake, as portrayed in

* We are glad to see by a notice published by a son of the late Dr. Drake, that the materials for the second volume of his great work on the Diseases, &c., of the Interior Valley, are left in such a condition that the volume will be published.

the discourse by Dr. Gross. The subjects were worthy the pen of the writer of the discourse. It will be read with interest, not only by the numerous friends of the deceased, but by all who are fond of enjoying, by means of biography, intercourse with the great and good among the dead, as well as cultivating the society of the excellent among the living.

